# Two case reports using a proposed oral risk assessment tool for older people near the end of life

**DOI:** 10.1002/cre2.566

**Published:** 2022-03-29

**Authors:** Kumi Tanaka, Takeshi Kikutani, Takashi Tohara, Shiho Sato, Yoko Ichikawa, Noriaki Takahashi, Fumiyo Tamura

**Affiliations:** ^1^ Division of Rehabilitation for Speech and Swallowing Disorders The Nippon Dental University Koganei Tokyo Japan; ^2^ Division of Clinical Oral Rehabilitation The Nippon Dental University Graduate School of Life Dentistry Koganei Tokyo Japan

**Keywords:** end of life, outcome, risk‐map, survival prognosis

## Abstract

**Objectives:**

We developed a prototype technique that expresses the need for intervention and the effectiveness of the treatment when “not being at risk of injury to the oral cavity or to general health” due to the presence of teeth or prostheses is taken as the desired outcome of dental treatment for older people near the end of life. The objective of this study was to use the prototype risk assessment matrix to identify the risk for each patient according to their course and show the effectiveness of treatment.

**Material and Methods:**

We produced a prototype Dental Risk Map (Dental R‐map) based on the risk map method of risk management. Risk is classified into three levels according to the level of tolerability: (A) Risk for which watchful waiting should be included among measures to be considered; (B) risk for which intervention should be considered; or (C) risk requiring urgent intervention.

**Results:**

We report the application of this technique to two men in their 80s. Both were assessed as risk tolerability Level C, requiring immediate intervention. Dental treatment eliminated this risk in one and reduced it to Level B in the other.

**Conclusions:**

We developed the prototype Dental R‐map to identify oral risks and indicate the need for intervention to address these risks and the effectiveness of treatment for older people near the end of life. We used the Dental R‐map for two patients and successfully avoided oral risks that might cause physical injury in both cases until their deaths.

## 1 INTRODUCTION

Improvements in dental and oral health mean that an increasing number of older adults people still have many of their own teeth. In Japan, since 1989, the Ministry of Health and Welfare and the Japan Dental Association have been promoting the so‐called “8020 movement” with the aim of people still having 20 or more of their own teeth at age 80 (Ishii, 2005; Shinsho, 2001). The goal is to be able to relish the enjoyment of eating with one second's own teeth throughout life since most foods can be chewed if people have 20 or more teeth. In a 2016 survey, this goal was achieved by 51.2% of those concerned (Hiroko Miura, 2019). However, the prevalence of dental caries is increasing and because more teeth are retained, severe periodontitis is now common among older people, and studies are now pointing out that these teeth require treatment (Batchelor, 2015; Hiroko Miura, 2019; Hoeksema et al., 2018; Kobayashi et al., 2016). The more remaining teeth that older people have, the greater the proliferation of salivary bacteria (Tohara et al., 2017) potentially causing fever (Shimazaki, 2011). Medical associations have also reported that a high proportion of foreign bodies found in the gastrointestinal tract and airway were teeth, lost fillings, other dental restorations, or small dentures (Mosca et al., 2001; Webb, 1995). These studies suggest that the presence of teeth or prostheses may increase the risk of accidents or injuries affecting the oral cavity or general health in older people receiving long‐term care who have difficulty attending outpatient appointments (Batchelor, 2015; Kobayashi et al., 2016).

Previous publications have reported perspectives on care at the close of life (Lynn, 2001) and trajectories of disability in the last year of life (Gill et al., 2010). In the process of functional decline as death approaches, deterioration of oral status and difficulties with dental treatment are anticipated to accelerate rapidly, rather than proceed in a linear fashion. The need for the medical community to shift its way of thinking from the current emphasis on extending life to a viewpoint that tolerates a diverse range of value judgments is now a subject of discussion at medical conferences. This means that a wide variety of options are now available in clinical settings, from prioritizing life extension to accompanying people as they die naturally, and this is giving rise to a range of different policies and declarations of stances (Detering et al., 2010; Guideline, 2015; Hall et al., 2011; Radbruch & Payne, 2009).

In the development of dentistry, a healthy aging society has been the desired outcome (Fukai, 2013). That is, the emphasis has been on masticatory function based on the presence of teeth. Current treatment policy formulated with this as the objective may not necessarily be appropriate for older people approaching the end of life. No policy addressing such older people has yet been published, and a new policy is therefore required. Furthermore, while narrative‐based medicine tends to be more important at this stage of life, objective indicators are also needed. In this study, we developed a prototype technique that expresses the need for intervention and the effectiveness of the treatment when “not being at risk of injury to the oral cavity or to general health” due to the presence of teeth and/or prostheses was taken as the desired outcome of dental treatment for older people near the end of life. The objective of this study was to use the prototype risk assessment method to identify the risk for each patient according to the course and show the effectiveness of treatment.

## 2 METHODS

A prototype Dental Risk‐Map (Dental R‐map) to express the need for intervention to address risks and the effectiveness of treatment was created as shown in Table 1. Risk maps (R‐maps) are a method of quantifying risk by using a matrix with the frequency of occurrence on the vertical axis and the extent of the injury on the horizontal axis to express the severity of the risk (Anang et al., 2016; Schiele et al., 2012; Zhao, 2012). They are widely used in economics and industry as visual representations of risky and safe domains, and they allow risks before intervention, the risk‐mitigating effect of the intervention, and postintervention risks to be plotted in the same matrix. In the Dental R‐map, the severity of oral risks is plotted on the horizontal axis and the probability of their occurrence on the vertical axis, each on a five‐grade scale. The severity is classed as: (1) None (transient discomfort); (2) Negligible (transient problem, injury not requiring specialist medical care); (3) Marginal (injury requiring specialist medical treatment); (4) Critical (potentially fatal risk, severe); or (5) Catastrophic (potentially fatal risk, very severe). The probability is classed as: (1) Improbable; (2) Remote; (3) Occasional; (4) Probable; or (5) Frequent. Based on this, the risk tolerability level is classed as: (A) Risk for which watchful waiting should be included among measures to be considered; (B) risk for which intervention should be considered; or (C) risk requiring urgent intervention. The risk level increases further to the right. The severity and probability are determined in light of survival prognosis, level of consciousness, functional status, environmental factors, and other factors to be taken into account in the dental treatment of older people. Interventions are performed based on the risk tolerability level, and the risk mitigation process and interventions implemented are summarized in a risk analysis matrix, as shown in Tables 1 and 2.

**Table 1:** Dental Risk‐Map

Severity→	1. None	2. Negligible	3. Marginal	4. Critical	5. Catastrophic
↓Probability	Transient discomfort	Transient problem, injury not requiring specialist medical care	Injury requiring specialist medical care	Potentially fatal risk (severe)	Potentially fatal risk (very severe)
5 Frequent	B	C	C	C	C
4 Probable	B	B	C	C	C
3 Occasional	A	B	B	C	C
2 Remote	A	A	B	B	C
1 Improbable	A	A	A	B	B

*Note*: The horizontal axis; the severity of oral risks, the vertical axis; the probability.

The risk tolerability level is classed as: (A) Risk for which watchful waiting should be included among measures to be considered; (B) risk for which intervention should be considered; or (C) risk requiring urgent intervention.

**Table 2:** Risk assessment table

			Risk analysis			Risk evaluation			Risk control			
Case ID			Hazard	Hazard situation	Harm	Severity	Probability	Risk	Verification measures	Severity	Probability	Risk
1	Dementia patient with involuntary jaw movements	Watchful waiting	Teeth luxation	Aspiration or accidental ingestion resulting from teeth falling out	Aspiration pneumonia, choking, surgical removal	4	5	C	Extraction	–	–	–
		Postextraction assessment (second examination)	Increased mobility due to occlusal trauma from remaining teeth	Aspiration or accidental ingestion resulting from teeth falling out	Aspiration pneumonia, choking, surgical removal	4	3	C	Careful monitoring of mobility	4	2	B
									Occlusal adjustment			
									Instruct care workers on bite block use			
		Reassessment (third examination)	Increased mobility due to occlusal trauma from remaining teeth	Aspiration or accidental ingestion resulting from teeth falling out	Aspiration pneumonia, choking, surgical removal	4	1	B	Careful monitoring of mobility	4	1	B
									Occlusal adjustment			
									Instruct care workers on bite block use			
		Other	Poor oral environment	A reservoir of respiratory tract infections	Fever, aspiration pneumonia	4	5	C	Professional oral care by dental professionals	4	4	C
									Instructing staff on oral care methods			
2	Patient with end‐stage bladder cancer	If reattached	Bridge loss, root caries	Aspiration or accidental ingestion resulting from reattached bridge falling out	Aspiration pneumonia, gastrointestinal perforation, surgical removal	5	2	C	Reattach using adhesive resin cement	5	1	B
									Anchor to fix adjacent teeth			
									Increase frequency of visits and check carefully for mobility			
		Reassessment (second examination)	After reattachment bridge, root caries	Aspiration or accidental ingestion resulting from reattached bridge falling out	Aspiration pneumonia, gastrointestinal perforation, surgical removal	5	1	B	Maintain frequency of visits and check or mobility	5	1	B
		Other	Poor oral environment	A reservoir of respiratory tract infections	Fever, aspiration pneumonia	4	3	C	Professional oral care by dental professionals	4	2	B
									Instruct families and hospital staff on oral care methods			

In this study, we used and discussed Dental R‐map in two cases. This study is a preliminary study. It is necessary to utilize the Dental R‐map as an objective tool to clarify oral risks and to express the effects of dental treatment in older people near the end of life.

## 3 CASE REPORTS

### 3.1 Case 1

**Figure 1:**
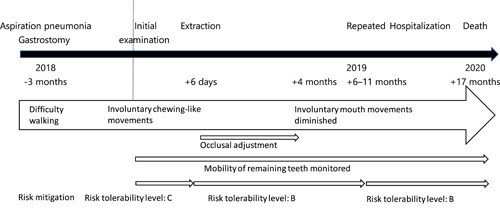
Timeline in Case 1

The case timeline is shown in Figure 1. A man in his 80s living in a nursing home requested an examination with a chief complaint of a mobile tooth in October 2018. His medical history included dementia, previous cerebral hemorrhage, and aspiration pneumonia. He had been hospitalized for aspiration pneumonia for 3 months before the initial examination. During this period of hospitalization, a gastrostomy tube had been inserted. Since then, he had gradually started exhibiting involuntary chewing‐like movements and turning his head. While an attendant was performing oral care after discharge from the hospital, some of the patient's incisors were subluxed when he bit down on a bite block; therefore, home‐visit dental treatment was started. The activities of daily living of the patient required total dependence, and his level of consciousness was Japan Coma Scale (JCS) III‐200.

**Figure 2:**
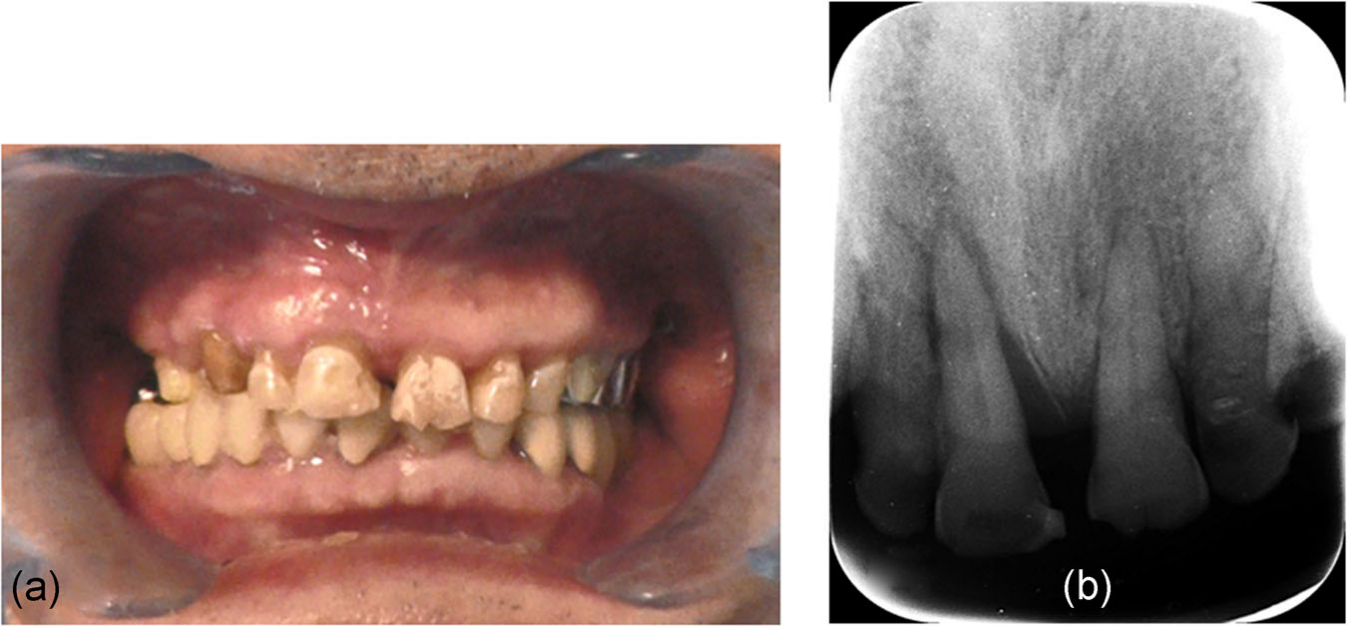
Oral fundings in Case 1; (a) intraoral anterior view, (b) intraoral radiograph

Oral findings showed third‐degree mobility of the upper right central and lateral incisors, which wobbled severely every time the patient opened and closed his mouth as shown in Figure 2. The occlusal condition was assessed as Eichner index B2. Occlusion of the left molars and incisors was maintained. The patient did not wear dentures. Membranous substances and oral halitosis were evident. The patient's oral hygiene was poor.

The general state of health: The patient's life expectancy was estimated at up to 2 years, in light of (1) his doctor's clinical judgment, (2) the fact that gastrostomy patients in Japan must be expected to survive for at least 2 years (Suzuki et al., 2012) and (3) the patient's history of aspiration pneumonia and dysphagia.

### 3.2 Treatment and course

Oral risks were assessed using the Dental R‐map as shown in Tables 1 and 2. On risk analysis, the hazard was considered to be teeth luxation, a situation in which teeth fall off, resulting in accidental ingestion or aspiration, choking, and potential body injury, including aspiration pneumonia or surgical removal. In risk evaluation, the severity was assessed as “critical (potentially fatal risk, severe).” The probability of occurrence during the patient's anticipated remaining 2 years of life was assessed as “frequent.” As a result, the risk tolerability level was considered to be “C: Risk requiring urgent intervention.” In terms of risk control, it was considered that this situation would be resolved by teeth extraction.

The postintervention residual risk was considered to consist of occlusal trauma to other remaining teeth due to the loss of occlusal support for the incisors. However, the implementation of occlusal adjustment and careful monitoring for risk control should reduce the probability of this risk, although its severity would be unchanged, lowering the risk tolerability level to “B: Risk for which intervention should be considered.” After consulting the patient's doctor, it was decided to extract the teeth concerned. Because the patient was incapable of expressing his own thoughts, consent was obtained from family members. The teeth were extracted by the usual method under local anesthesia. As postextraction risk control, we instructed the nursing home staff to place the bite block between the molars rather than the front teeth when carrying out oral care. Occlusal adjustment and careful monitoring during frequent visits continued to be carried out as scheduled.

In addition, “hazard other than the chief complaint” included deterioration of the oral environment. A hazard situation was considered to be a reservoir of respiratory infections. Fever and aspiration pneumonia were considered harmful. In risk evaluation, the severity was assessed as “critical.” The probability of occurrence in the next 2 years, which was the expected life prognosis, was determined to be “frequent.” As a result, the risk tolerability level was judged to be “C: Risk requiring urgent intervention.” As risk control measures, oral care by dental professionals and oral care instructions for the nursing home staff were planned. Since the patient's general condition was still poor, the risk severity was considered “critical” and the risk tolerability level was assessed as “C: Risk requiring urgent intervention.” As the frequency and quality of oral care improve, the probability is likely to reduce.

During the following year, the patient was hospitalized multiple times for bronchitis and pneumonia. The repeated jaw movement of gradual opening and closing diminished from around 4 months after tooth extraction and had disappeared almost completely by 10 months after the extraction. A reassessment of the Dental R‐map for the remaining teeth found no change in severity, which was still “critical,” but the probability had decreased to “1. Improbable,” and the risk tolerability level was considered to be “B: Risk for which intervention should be considered.” In response to the deterioration of the oral environment, oral care was continued by dental professionals and nursing home staff. The risk tolerability level was elevated to “C: Risk requiring urgent intervention.” A further year later (1 year 4 months after extraction), the patient died in the nursing home. Tooth loss and other risks had been successfully avoided until the end of life. Fever and respiratory infections could not be completely avoided.

### 3.3 Case 2

**Figure 3:**
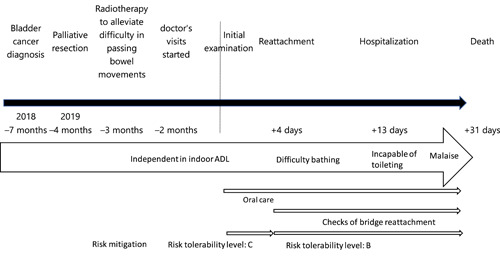
Timeline in Case 2

The case timeline is shown in Figure 3. A man in his 80s living at home was first examined in July 2019. His chief complaint was that the bridge fell out. He had been diagnosed with bladder cancer the previous year. Due to his advanced age, highly invasive treatment was judged to be undesirable, and he was receiving only palliative therapy. In May 2019, he had started receiving care at home. In July, his prosthesis fell out, and his home‐visit doctor requested home‐visit dental treatment. The patient was almost independent in terms of indoor activities of daily living, but he spent most of his time resting in bed. His cognitive function was within the normal range for his age, and his level of consciousness was JCS I‐1.

**Figure 4:**
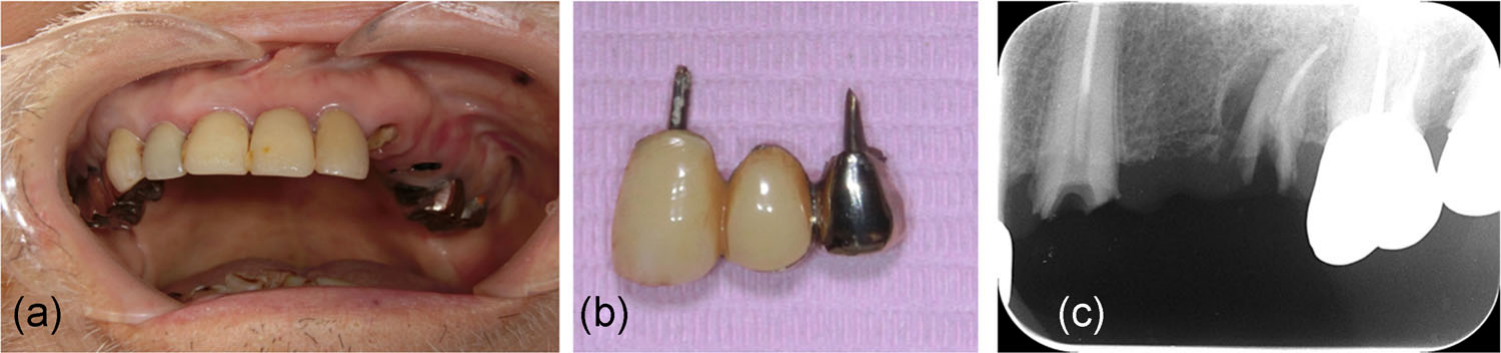
Oral findings in Case 2; (a) intraoral view of the upper jaw, (b) detached bridge, (c) intraoral radiograph

Oral findings showed that a bridge covering the left upper teeth 3, 4, and 5 had fallen off, and that root caries were present as shown in Figure 4. This was the first time this bridge had fallen off, and the patient requested that it be reattached. The Eichner index was A‐2. Although the patient was independent in his oral cleaning and the clean status was normal, he had xerostomia, and tongue coating was evident. As such, it was expected that his oral problems would increase in the near future.

The general state of health: The patient's life expectancy was estimated at about 1 month, in light of (1) his doctor's clinical judgment and (2) the fact that palliative prognostic index (PPI) score (Morita et al., 1999) was 6.0.

### 3.4 Treatment and course

Oral risks were assessed using the Dental R‐map as shown in Tables 1 and 2. On risk analysis, it was confirmed that the bridge had fallen off because of root caries. The hazard situation generated by its reattachment included aspiration or accidental ingestion should it again fall out. Potential harms included aspiration pneumonia, gastrointestinal perforation, or surgical removal. In risk evaluation, the severity was classed as “catastrophic (potentially fatal risk, extremely severe)” because the patient was spending more time sleeping and his activities of daily living would further diminish in his final days. The probability of occurrence during the patient's anticipated remaining lifespan was assessed as “2. Remote.” As a result, the risk tolerability level was considered to be “C: Risk requiring urgent intervention.” Accordingly, reattaching the bridge was not recommended. However, the patient requested that it be reattached and since the bridge included the front teeth, it was considered that the patient's postmortem facial appearance should also be taken into consideration. As risk control, if the detached bridge was reattached using adhesive resin cement and also fixed to the adjacent teeth, and frequent visits were made to check it, this should reduce its probability of falling out, although its severity would be unchanged, lowering the risk tolerability level to “B: Risk for which intervention should be considered.” Informed consent to this treatment was obtained from the patient and his family. The bridge was reattached as described above. As postintervention risk control, careful monitoring during frequent visits continued to be carried out as scheduled.

Other than the chief complaint, hazards included deterioration of the oral environment. A hazard situation was considered to be a reservoir of respiratory infections. Fever and aspiration pneumonia were considered harmful. In risk evaluation, the severity was assessed as “critical.” The probability of occurrence during the patient's anticipated remaining lifespan was assessed as “3. Occasional.” The risk tolerability level was judged to be “C: Risk requiring urgent intervention.” As a risk control, we planned to provide oral care by dental professionals and guidance on oral care methods by family members. Although the severity remained unchanged, the probability was reduced. Thus, the risk tolerability level was estimated to be “B. Risk for which intervention should be considered.”

**Figure 5:**
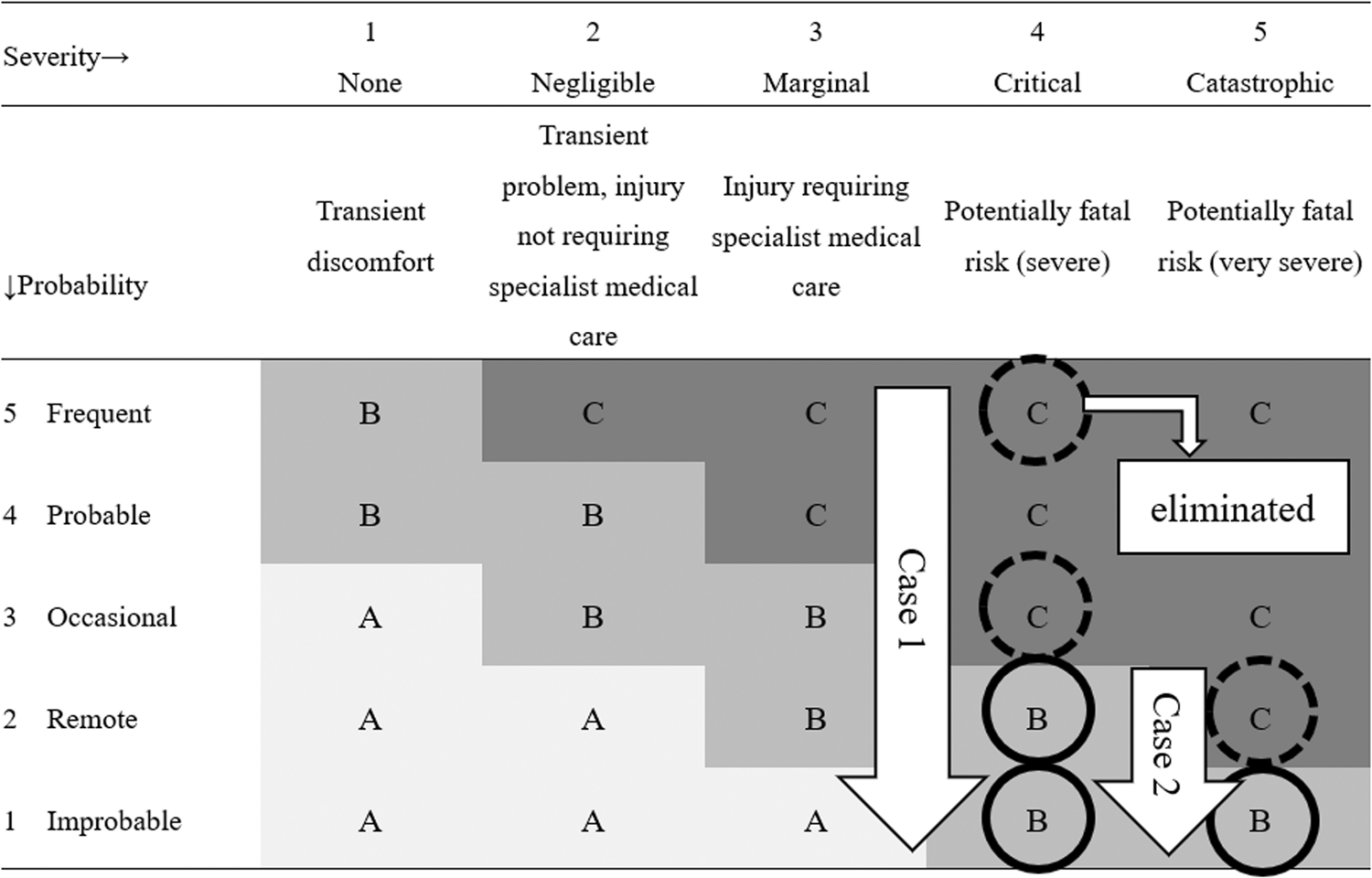
The risk mitigation for each case in Dental R‐map. Case 1: The risk mitigation for mobility teeth. Case 2: The risk mitigation for falling out bridge. The horizontal axis; the severity of oral risks, the vertical axis; the probability. The risk tolerability level is classed as: (A) Risk for which watchful waiting should be included among measures to be considered; (B) risk for which intervention should be considered; or (C) risk requiring urgent intervention. Dental R‐map, Dental Risk Map

The patient was unable to take a bath 12 days after the initial examination and incapable of toileting 13 days after the initial examination; at that time, he was admitted to the hospital where his home‐visit doctor was employed. A visit was made to the patient on the hospital ward for dental treatment, and the Dental R‐map was re‐evaluated. Both the severity and the probability of reattachment bridge were unchanged, and the risk tolerability level was B. Frequent visits were continued. Due to terminal cancer, deterioration of the oral environment emerged, although oral care by dental professionals, family members, and hospital staff was appropriate. The patient died 31 days after the initial examination. Bridge detachment and other risks were successfully avoided through the end of life. Fever and aspiration pneumonia were also prevented. The risk mitigation for each case in the R‐map is shown in Figure 5.

## 4 DISCUSSION

We produced a prototype Dental‐R map and used it to express the need for intervention to address oral risks and the effectiveness of treatment in older people near the end of life.

In Case 1, the patient had incisor luxation. In light of his mid‐to‐long‐term life expectancy, the risk tolerability level of teeth falling out was considered to be “C: Risk requiring urgent intervention.” This risk was eliminated by extraction. The postintervention risk tolerability level was “B: Risk for which intervention should be considered,” meaning that risk mitigation was feasible. The patient's general condition changed as his course progressed, and on reassessment, the probability level had decreased.

In Case 2, the patient's bridge, which included his canines, had fallen out. Since he was not expected to live for long and the risk tolerability level of reattachment was C, our judgment was that it should not be reattached. However, at the patient's own request and in consideration of the effect on his postmortem facial appearance, verification measures were implemented by firmly reattaching and careful checking during frequent visits, reducing the risk tolerability level to B. The treatment provided, reattachment, would not normally have been recommended, but risk mitigation measures were put in place. In both cases, we were able to demonstrate risk elimination and risk mitigation as the effectiveness of treatment.

The R‐map used in this study is one method of risk assessment. Risk assessment techniques include numerical approaches, integration, matrix, and risk graph methods. The R‐map is a matrix method in which a matrix is constructed with the severity of the injury and the probability of its occurrence are plotted on the vertical and horizontal axes, and the risk level is determined by the allocation of risk indices to cells in the matrix depending on the combinations of levels of each parameter. It has the advantage that it enables a simple comparison of risks before and after the use of risk mitigation measures, and we produced the prototype Dental R‐map because of this simplicity. In the medical field, an R‐map for patients with chronic kidney disease is already in use (Kidney Disease: Improving Global Outcomes KDIGO CKD Work Group, 2013).

In this study, the Dental R‐map was used in two cases. Its first advantage is that it can indicate the need for intervention. In older people, the risk of postextraction hemorrhage or poor general condition is a reason for not actively performing a dental treatment. However, using the Dental R‐map for advanced risk assessment shows the need for intervention. This enables alternatives to be explored if the most recommended treatment cannot be performed for reasons such as the risk of postextraction hemorrhage, poor general condition, the wishes of the patient and their family, or environmental factors. In Case 1, such alternatives might have included fixing the mobile teeth or crown milling. This contributed to the early removal of the teeth that would normally have required treatment and the consequent mitigation of the risk of physical injury. At the same time, the same process used in the original risk assessment of risk analysis, risk evaluation, and risk control must be conducted for the postintervention residual risks, with interventions performed so as to avoid injury from occurring as far as possible.

Other possible hazards include pain due to dental caries, tooth mobility due to periodontitis, tooth fracture or dislocation, prosthetic dehiscence, mucosal disease, deterioration of the oral environment, ill‐fitting denture, and dysphagia. Hazard situations include aspiration due to tooth or prosthesis falling out, accidental ingestion, a reservoir for respiratory infections, difficulty in feeding, and so forth. Resulting harms include fever, choking, aspiration pneumonia, and low nutrition. These hazards can be addressed in the same way.

The second advantage of the Dental R‐map is that it identifies the effectiveness of treatment. Conventionally, dentistry has developed with the aims of avoiding tooth loss and restoring occlusion in the event of defects. Studies of tooth numbers have found that a larger number of remaining teeth results in a better occlusal force, physical function, nutritional status, and dietary diversity. Occlusal support has similarly been associated with masticatory ability, skeletal muscle, dementia, and frailty. However, in older people approaching the end of their lives, the presence of teeth or prostheses may cause the risk of injury to the oral cavity or to general health in the form of bite wounds, fever, accidental ingestion, aspiration, or gastrointestinal foreign bodies (Batchelor, 2015; Kobayashi et al., 2016; Mosca et al., 2001; Shimazaki, 2011; Tohara et al., 2017; Webb, 1995). Previously, there was no means of expressing the effectiveness of such treatment at the point in a person's life when restoring occlusion or preserving the number of teeth no longer contributed to their health (Batchelor, 2015; Kobayashi et al., 2016; Shimazaki, 2011; Suzuki et al., 2015; Tohara et al., 2017). The reduction in risk tolerability level indicated by the Dental R‐map is a contribution to expressing the effectiveness of treatment in older people near the end of life.

In our two cases, the patients had poor oral environment as a symptom other than the chief complaint. Deterioration of the oral environment is common in older adults near the end of life. A poor oral environment increases the hazard of respiratory tract infections, leading to fever and aspiration pneumonia (Bassim et al., 2008; Yoneyama et al., 1999) In Case 2, the probability of improvement was not estimated to be high because the patient had terminal cancer that significantly impacted on his prognosis. In Case 1, both the severity and probability of fever and aspiration pneumonia were high due to the patient's poor general condition. The risk tolerability level was also high. In both cases, oral care instructions provided to the caregivers were effective. Considering the limited manpower and substantial care burden, patient support was sufficient. There are many clinical situations where the primary focus should be placed on reducing the deterioration of the oral environment. It has already been reported that oral care reduces the risk of fever and pneumonia. As a risk control, dental professionals need to properly explain the patient's oral status to caregivers and instruct them on how to clean the teeth, mucous membranes, and dentures, to reduce the risk tolerability level for fever and aspiration pneumonia as much as possible. The risk tolerability level can be reduced by educating and improving the understanding of caregivers on quality oral care, as well as receiving treatment from dental care professionals.

This study, the Dental R‐map, and R‐maps in general have several limitations. The first is that only a limited number of types of risk parameters can be used in R‐maps. This means that parameters, such as survival prognosis, level of consciousness, activities of daily living, environmental factors, and the patient's wishes cannot be used as individual parameters. Although consideration of these factors is essential for the dental treatment of older people being cared for either at home or in an institution, the diversity of patients' functional status and environments makes the quantification of individual factors unrealistic. Severity and probability must therefore be determined at the discretion of dental professionals. The potential for individual experience or judgments to affect the determination of severity and probability is a matter of concern, and measures must be taken to prevent errors in the overall judgment of risk tolerability level by the dentists who provide home‐visit dental treatment. The second limitation is the difficulty of predicting life expectancy, which is a determining factor for the level of probability. Although predictions can be made on the basis of disease or the requirement for tube‐feeding, these range widely in different countries and reports. Doctors' estimates of the life expectancy of patients with advanced cancer are known to be inaccurate (Glare et al., 2003; Taniyama et al., 2014). The PPI (Morita et al., 1999) or Palliative Prognosis Score (Glare & Virik, 2001) is used for predicting short‐term prognosis, and they are of demonstrated value, but mid‐to‐long‐term survival prognosis is difficult to predict, and in‐depth discussions with the patient and the doctor, family members, and other medical professionals are required to use the Dental R‐map. The third limitation is that further studies are required to quantify the timing and frequency with which the Dental R‐map should be used for risk assessment. Changes in the patient's status, such as changes in the state of consciousness, tooth clenching or grinding, or the appearance or diminution of involuntary movements, as well as changes in other factors, such as caregivers or living environment, may be the right time to reassess the Dental R‐map. Sufficient clinical evaluation and further studies involving the use of the Dental R‐map in larger numbers of patients with a range of different diseases or conditions and characteristics are required to investigate the timing of reassessment and what sort of treatment is carried out at each stage.

## 5 CONCLUSIONS

We developed the prototype Dental R‐map to identify oral risks and indicate the need for intervention to address these risks and the effectiveness of treatment for older people near the end of life. We used the Dental R‐map for two patients, and oral risks that might cause physical injury were successfully avoided in both cases until their deaths. Dental treatment should reduce the possibility of oral or general physical injury to help people spend their final days without distress. Sufficient clinical evaluation is essential for the widespread use of the Dental R‐map to achieve the request.

## AUTHOR CONTRIBUTIONS

Kumi Tanaka, Takeshi Kikutani, and Fumiyo Tamura conceptualized the methods and administered the project. Kumi Tanaka, Takeshi Kikutani, Takashi Tohara, Shiho Sato, Yoko Ichikawa, Noriaki Takahashi, Fumiyo Tamura wrote, reviewed, and edited the manuscript. Kumi Tanaka, Takeshi Kikutani, Fumiyo Tamura, Takashi Tohara, and Noriaki Takahashi validated the methods. Kumi Tanaka wrote the original draft.

## CONFLICTS OF INTEREST

The authors declare no conflicts of interest.

## ETHICS STATEMENT

This study was conducted after obtaining approval from the Nippon Dental University School of Life Dentistry Ethics Committee (approval code: NDU‐T2020‐13). Written informed consent was obtained for all participants. If the participant had severe dementia, informed consent was obtained from their family members or caregivers.

## Data Availability

Data that support the findings of this study are available from the corresponding author upon reasonable request.
